# Data on inter-locking nail in humeral shaft fractures among Indian patients

**DOI:** 10.6026/97320630018811

**Published:** 2022-09-30

**Authors:** Amit Lakhani, Ena Sharma, Amit Kapila, Sandeep Singh

**Affiliations:** 1 Dr BR Ambedkar State Institute of Medical Sciences, Mohali, Punjab, India; 2 Maharishi Markandeshwar College of Dental Sciences and Hospital Mullana, Ambala Haryana, India; 3 Fortis Healthcare, Mohali, Punjab, India

**Keywords:** Interlocking nails, fractures, close reduction

## Abstract

Humeral shaft fractures are commonly seen orthopaedics injuries. Open reduction with internal fixation (ORIF) with plating is a gold standard procedure despite various issues such as infection, radial nerve palsy and no-nunion. Close reduction with interlocking nails (ILN) is not a very popular procedure. Therefore, it is of interest to collect data on the significance of interlocking nail in different pattern of humerus shaft fracture. 30 patients with closed humeral shaft fracture participated in this study. The fractures were classified according to their descriptive location as proximal, middle and distal. All surgeries were performed by a single surgeon familiar with the ILN procedure. All patients had appropriate clinical, radiological and pre and postoperative assessment. Data on patients were collected at 2, 6weeks, 12weeks, 18 weeks and 6 months. 19 cases with middle third and distal third fractures were united within 10-14 weeks. 6 cases of proximal shaft fracture were united in 14-18 weeks. According to Rodrı ´guez–Merchant criteria - Middle shaft fracture has shown good results (n=9, 75%) followed by distal third shaft fracture (n=6, 60%) and proximal third fracture (n=1, 12.5%). Though there is decrease in mean ASES score in all three groups of fractures but the Mid shaft fracture has shown significant decline in ASES score suggesting improvement in pain and ROM after 6 months. Thus, ILN of humerus is a simple and a safe procedure for treating fractures of middle and distal third shaft humerus. However, this study does not support ILN for the management of proximal third humerus fracture.

## Background:

The prevalence rate of Humeral shaft fractures represents 3-5% of all managed fractures [1]. The surgical intervention includes open reduction with internal fixation or close reduction with interlocking nails [2]. Open reduction with internal fixation (ORIF) always remains the gold standard, but issues recognized during this method are radial nerve injury, excessive soft tissue stripping, difficulty with complex fracture patterns, and the risk of mechanical failure in osteopenic bone [3]. Close reduction with interlocking nails (ILN) is not a very popular procedure due to very few long-term longitudinal studies and technical difficulties. But it has many advantages such as periosteum-sparing stabilization of complex fractures, as the surgery does not involve periosteal stripping and reaming produces act as an auto graft [4]. It is a mini-invasive procedure with improved biomechanics and load-bearing feature of the implant. Fractures managed with ILN have better chances of union, lesser blood loss and lesser hospital stay. Hence, this leads to an increased interest in using the humeral intramedullary nail for treatment after evaluating the success rate associated with nailing in other long bones. With growing advantages in technology, improvement in implant design, the possibility of antegrade insertion, rotational stiffness, and growing experience in surgical technique have led to better results [5]. Therefore, this study was conducted to evaluate and compare the radiological and functional outcome of ILN in proximal, middle, and distal third humerus fracture.

## Methodology:

This retrospective clinical study of management of humeral shaft fractures by antegrade interlocking nail fixation from December 2017 to December 2020 was conducted in the department of orthopedics in MMMCH, Kumarhatti, Solan in which data of 30 patients with a mean age of 39.36 (Male 80% and 20% Female ) of humeral shaft fracture was considered based on:

Inclusion criteria:

[1] Humeral shaft fracture which required operative intervention 

[2] Age of patient 18 years or more 

Exclusion criteria: 

[1] Pathological fractures 

[2] Segmental fractures 

[3] Radial nerve injury following closed reduction 

[4] non-cooperative patients 

[5] fractures within 4cm of the proximal and distal end of the humerus 

The fractures were classified according to their descriptive location. All surgeries were performed by single surgeon, familiar with the procedure of ILN. Patients had appropriate clinical, radiological, and preoperative assessment before operative intervention.

## Surgical procedure:

Antegrade interlocking nailing was done using bent nail design (Russell-Taylor type, Sharma) in a supine position in all patients. The antero lateral approach was used for the entry point of the nail. Make an incision diagonally from the antero lateral corner of the acromion, splitting the deltoid in line with its fibers in the raphe between the anterior and middle thirds of the deltoid. Under direct observation, incise the rotator cuff in line with its fibers. The nail insertion site lies on the axis of the humeral shaft. It is typically near the highest point of the humeral head. It is slightly anterior to the center of the greater tuberosity. A supra spinatus split is necessary to access the area. When reaming is complete, pass the nail down the humeral canal, avoiding the distraction of the fracture; ensure that the nail is below the articular surface of the humeral head. Distal locking was done using the free hand technique under the image intensifier. Proximal locking was done after careful spreading of soft tissue for taking care of the axillary nerve. Supra spinatus split was repaired before closure. The patient arm was supported with a simple neck sling postoperatively. Passive flexion and extension were started to encourage a range of motion (ROM) according to pain tolerance after the first postoperative day. Early Rehabilitation is not recommendable with conservative and plating procedures but in ILN procedure it was performed postoperatively from day one to avoid stiffness in joints. Patients were followed up at 2, 6weeks, 12weeks, 18weeks and 6 months. Major consideration was given to the restoration of ROM in the shoulder and elbow. The pain was analyzed by VAS Score, range of motion of elbow and shoulder according to an American Shoulder and Elbow Surgeons (ASES) score for 13 activities of daily living requiring the shoulder and elbow movement with each activity carrying a maximum of 4 points. Operative time was also recorded and another scale called Rodrıguez-Merchant criteria (Table 1 - see PDF) was used to reach the outcome of the study population. To observe the signs of union and callus formation radiological analysis was done with AP and Lateral view in each follow-up.

## Results:

In this study of 6 months follow-up, 30 cases of humeral shaft fractures treated by ILN were evaluated. The study population consisted of 24(80%) males and 6(20%) females, with a mean age of 39±33 (19-72years) Middle third shaft fracture (n=12, 40%) was most frequently affected followed by distal one-third (n=10, 33.3%) and then proximal one-third humeral shaft fracture (n=8, 26.6%). The operative time was also analyzed and it was observed that maximum mean operative time was taken for proximal shaft fracture followed by middle third and minimum time taken with a mean of 45minutes for operating distal shaft fracture. Union was defined as the presence of bridging callus in two planes and the absence of pain and movement at the fracture site. Out of 30, 19 cases of the middle third and distal third fractures were united within 10-14 weeks. 6 cases of proximal shaft fracture were united in 14-18 weeks. There was no significant difference seen between middle third and distal third shaft fracture with respect to time of union in weeks, but if compared to proximal shaft fracture there was significant clinical difference observed (when compared with both groups P<0.05). Non-union was seen in 2 cases of proximal third fracture and 1 case of middle third fracture (Table 2 - see PDF). Functional results of the study were evaluated by Rodrı ´guez - Merchant criteria and it was seen that middle third fracture has shown excellent results with (n=9, 75%) followed by distal third shaft fracture (n=6, 60%) and proximal third fracture (n=1, 12.5%). Poor results were seen more (n= 3, 37.5%) in proximal third shaft fracture. There was a significant difference seen between functional outcome among groups with middle third fracture vs distal third fracture vs proximal third fracture (P<0.01) (Table 3 - see PDF). ASES scoring was done at each visit during follow up and it was critically evaluated by asking 13-point questions. In this study though there was a decrease in mean ASES score in all three groups of fractures but the mid shaft fracture has shown a significant decline in comparison among the fracture groups in ASES scoring which indicate excellent improvement concerning pain and ROM after 6 months. Whereas the proximal third fracture has shown the least improvement in the ASES score. There was a significant difference observed in mean ASES score among the Middle third fracture group Vs Proximal third fracture group ( P<0.01) and Distal third fracture vs Proximal third fracture group (P<0.05) and no significant difference was observed among Middle third and Distal third fracture groups. Main complication were non union in four cases (3 proximal, 1 middle), radial nerve palsy (one case), impingement syndrome (one case) and implant failure (one case). Impingement syndrome was seen in the case of a short stature female, in which the tip of the nail was protruding slightly. It was removed after 8 months of surgery after achieving union. In implant failure, the nail was removed and converted to a long plate with a bone graft. Union was achieved in 12 weeks in this case.

## Discussion:

Conservative management is the mainstay of management in humerus fracture. Small amount of shortening that cause minimal functional deficit is well tolerated by the patient [6]. Operative intervention is indicated in unacceptable reduction (shortening >3 cm, rotation > 30 degree, angulation >20 degree), polytrauma and open fracture [7]. The goal of operative intervention is the restoration of alignment with stable fixation to allow early motion and functional recovery. Choices are plate fixation, ILN, and external fixator (Ex Fix). Thus, Ex Fix is recommended for high energy gunshot wounds, fractures with significant soft tissue injuries, and fractures with massive contamination [8]. Plate osteo synthesis is a standard treatment for all humerus fractures. Some case series reported union rate up to 95% after plate fixation of humerus shaft fracture [9-10]. But plate fixation requires extensive soft tissue dissection, risk of radial nerve palsy and refractures after implant removal. In 1986 Brumback for first time reported the use of rush nails and ender nail in the management of humeral shaft fracture in polytrauma patients and reported a 94 %of union and a 62 % rate of excellent clinical results [11]. Gallagher et al. (1988) used a threaded nail in fixation of proximal and shaft fracture humerus in 33 patients and achieved excellent results in middle shaft fracture and satisfactory results in proximal fracture [12]. After this, few more studies have reported the advantage of locked intramedullary nailing in the management of humeral shaft fracture [13-15]. Non disturbance of fracture hematoma, soft tissue, and periosteum around the fracture with closed un-reamed nailing are the determinants for high rates of union and good results. Decreased risk of radial nerve palsy16 and use of locking bolt for rotational stability [17] are other positive factors in favour of ILN in humerus fracture.

By looking at all these factors, we used intramedullary nailing in treating humerus shaft fracture, in which conservative management was not possible. Male predominance in our study attributed to more outdoor activity of males as compared to females. The average mean age of 39.36 years represents bimodal age distribution, both in young patients (due to high energy trauma) and older patients (due to osteoporosis). In our study, we found a maximum number of cases in the middle shaft followed by distal and proximal shaft fracture. According to our best knowledge, this is the first study done to determine the outcome of ILN among the patterns of humeral shaft fracture. The mean operative time was maximum for proximal shaft and minimum for distal shaft fracture is because of the anatomical proximity of fracture. We achieved union in 27 cases of shaft fracture which is comparable to other studies in literature [18-19]. We got nonunion in 3 cases ofproximal humeral fracture, which may be due to mal-reduction as short proximal segment has wide medullary canal in proximal area which makes it difficult to achieve good reduction and stable fixation. Another factor responsible for nonunion is excessive mechanical stress in the area of humerus due to insertion of the deltoid muscle in proximal humerus. One of the nonunion case got implant failure and managed with open reduction and bone grafting. Nonunion in one case of the middle third shaft humerus was due to the large diameter of the nail causing distraction. Radial nerve palsy was seen in a single case of distal third humerus fracture, which recovered spontaneously in 12 weeks. Impingement syndrome due to nail migration was seen in one case of proximal fracture humerus and recovered full function after implant removal (after achieving union). Functional results of the study were evaluated by Rodrı ´guez - Merchant criteria [20], which showed the best result in the middle and distal third fracture. Poor result in the proximal fracture is seen in a majority of cases due to restriction of shoulder movement and vague pain in the shoulder area. In ASES scoring, we achieved better results in the middle and distal third fracture than the proximal group.

## Conclusions:

ILN of the humerus is a simple and safe procedure for treating fractures of the middle and distal third shaft humerus. Damage to vulnerable tissue in the proximal area can be avoided by minimal use of a drill bit. Un-reamed solid nails of small diameter should be used to prevent damage to the endosteal blood supply and to avoid distraction at the fracture site. The nail must be buried deep in the head to avoid impingement. The results of our study conclude that management of distal and middle shaft fracture has shown significant outcome with ILN because this is the minimally invasive procedure which maintains good blood circulation and implant stability hence leads to early callus formation and healing.
